# Supplementary material for: Intrasexual cuticular hydrocarbon dimorphism in a wasp sheds light on hydrocarbon biosynthesis genes in Hymenoptera
Source: Commun Biol. 2023 Feb 3;6:147. doi: 10.1038/s42003-022-04370-0 (PMC9898505; doi:10.1038/s42003-022-04370-0)
Supplement: Supplementary file 2 — Description of Additional Supplementary Files [file 42003_2022_4370_MOESM2_ESM.pdf]

# Description of Additional Supplementary Files

**File name:** Supplementary Data 1
**Description:** Odynerus spinipes genes significantly differentially expressed in batch 1-females with different chemotype. The table shows the gene IDs, the log2-fold change values (values < 0 indicate higher expression of genes in females with chemotype 2 than in females with chemotype 1), the p-value obtained with DESeq2, the adjusted p-value from applying the false discovery rate (FDR), and the predicted function of the genes based on homology.

**File name:** Supplementary Data 2
**Description:** Odynerus spinipes genes significantly differentially expressed in batch 2-females with different chemotype. The table shows the gene IDs, the log2-fold change values (values < 0 indicate higher expression of genes in females with chemotype 2 than in females with chemotype 1), the p-value obtained with DESeq2, the adjusted p-value from applying the false discovery rate (FDR), and the predicted function of the genes based on homology.
