# Supplementary material for: Intrasexual cuticular hydrocarbon dimorphism in a wasp sheds light on hydrocarbon biosynthesis genes in Hymenoptera
Source: Commun Biol. 2023 Feb 3;6:147. doi: 10.1038/s42003-022-04370-0 (PMC9898505; doi:10.1038/s42003-022-04370-0)
Supplement: Supplementary file 3 — Supplementary Data [file 42003_2022_4370_MOESM3_ESM.pdf]

Supplementary Methods

Trap nests

Odynerus spinipes trap nests consisted of 20 cm long plastic pipes with a clear diameter of 10 cm filled with eolian silt deposit. The eolian silt deposit contained pre-built 7 cm long channels with a diameter of 0.6 cm each. The trap nests were mounted on posts at a height between 5 and 100 cm from the ground in Büchelberg, Germany (49.027985, 8.164801) in February 2015. The prepupae were removed from the trap nests in March 2016 and stored in separate transparent gelatin capsules (empty hard gelatin capsules size 3, LUTOR trading & distribution, Cologne, Deutschland and Birmingham, United Kingdom). Each gelatin capsule was pierced with an insect needle to improve gas exchange. The prepupae were stored at 4 °C within a box containing a wet tissue (changed every two days). Synchronized development was triggered end of April 2016 by increasing the ambient temperature to 23 °C. This resulted in most males hatching one week and most females hatching two weeks later.

O. spinipes females used for transcriptome sequencing were collected from trap nests placed in Bad Muskau (51.548200, 14.714900; in 2017), Büchelberg (49.027985, 8.164801; in 2015 and 2017), and Eichenzell (50.495927, 9.704589; in 2016), and treated as described above.

Gas chromatography and mass spectrometry

CHC extracts of *Odynerus spinipes* samples kept in the laboratory (from Büchelberg [2015 and 2016] and from Eichenzell [2016]) as well as of the three wasps sampled at the field site Büchelberg in 2016 were processed with an Agilent 6890 gas chromatograph (GC) coupled to an Agilent 5975 mass selective detector (MS, Agilent Technologies, Waldbronn, Germany). The GC was operated in splitless injection mode and fitted with a DB-5 Fused Silica capillary column (30 m × 0.25 mm ID; film thickness: 0.25 µm; J&W Scientific, Folsom, United States). CHC extracts of wasps sampled in 2017 in the field at the field site Büchelberg were processed with HP series 6890 gas chromatograph (Hewlett Packard, Palo Alto, California, USA), equipped with a DB-5 column (30 m x 0.25 mm film thickness: 0.25 µm; J&W Scientific, Folsom, USA), coupled to a HP series 5973 quadrupole mass spectrometer (Hewlett Packard, Palo Alto, California). CHC extracts of wasps studied at the field site Tenneville in 2017 were analyzed with a gas chromatograph-flame ionization detector (Shimadzu GC-2010 system, Shimadzu, Wemmel, Belgium) equipped with a SLB-5 MS non-polar capillary column (5%-phenyl)-methylpolysiloxane stationary phase; 30 m × 0.25 mm ID; film thickness: 0.25 µm). We consistently applied the following temperature profile when analyzing CHC extracts of wasps: start temperature 60 °C, temperature increase of 20 °C per minute up to 150 °C, followed by a temperature increase of 5 °C per minute up to 300 °C, which was maintained for 10 min. Honey bee CHC extracts were analyzed with the Agilent GC-MS specified above and applying the following temperature profile: start

temperature 60 °C, followed by a temperature increase of 5 °C per minute up to 300 °C, which was maintained for 10 min.

Genomic DNA NGS libraries

We generated three genomic DNA NGS libraries from a single pool of DNA extracted from meso- and metasomas of ten males (collected May 15 and 16, 2013) and 31 females (unidentified chemotypes, collected May 15 and 16, 2013) of *Odynerus spinipes* collected near Würzburg, Germany (49.77977, 9.97065) (voucher samples of the female samples are available in the BioBank of Oliver Niehuis at the University of Freiburg: ON_0890–0899, ON_0923, ON_0924, ON_2277–ON_2295).

Raw read processing

All raw reads of genomic DNA were processed with trimmomatic[1] version 0.33, selecting paired-end mode (if applicable), the Illumina TruSeq3 adapter sequences provided with trimmomatic, and the parameters: LEADING:3, TRAILING:3, SLIDINGWINDOW: 4:25, MINLEN:50. The RNA-seq raw reads were processed with the same software.

Masking of transposable elements and annotation of protein-coding genes

We used RepeatModeler version 1.0.8 (http://www.repeatmasker.org) to compile a *de novo* repeat library. The pipeline identifies transposon (TE) nucleotide sequences by

comparing the *de novo* repeat library to entries in the NCBI nr database (downloaded 03/17/2016 from https://ftp.ncbi.nlm.nih.gov/blast/db/) and removing nucleotide sequences that are not associated with transposons. We applied the same filter parameters as used by Petersen et al.[2]. The pipeline further combines the filtered repeat library with the Metazoa-specific section of RepBase[3] version 20140131 to create the final repeat library. RepeatMasker[4] version 4.0.5 was then used to annotate TEs in the *O. spinipes* genome assembly and to generate a soft-masked version of the draft genome. Protein-coding genes were annotated with the BRAKER[5] *ab initio* gene prediction pipeline version 2.1. To this end, we first mapped the available trimmed RNA-seq raw reads onto the soft-masked version of the *O. spinipes* draft genome assembly using HISAT2[6,7] version 2.1.0 and converted the output into a sorted BAM file using SAMtools[8] version 1.7 and BAMtools[9] version 2.3.0. The resulting BAM file was, together with the soft-masked draft genome assembly, provided as input to the BRAKER2 pipeline (internally using GeneMark[10] version 4.33; Augustus[11] version 3.3; NCBI BLAST+[12,13] version 2.6.0; SAMtools[8] version 1.7; and BAMtools[9] version 2.5.1) to predict protein-coding genes. BRAKER2 parameters (apart from path specifications) were set as follows: --UTR=off --gff3 --softmasking. UTR annotation was disabled, as it was at the time of execution according to the documentation of Augustus 3.3 not suitable for annotation of non-model insect genomes.

Gene tree taxon sampling

We searched the proteomes of the following 36 Euarthropoda for genes/proteins of gene families of interest: *Acromyrmex echinatior* (OGS version 3.8[14]), *Aedes aegypti* (gene set version AaegL3.3[15]), *Amyelois transitella* (gene set version 1; NCBI GCA_001186105.1), *Apis mellifera* (OGS version 3.2[16]), *Bombus terrestris* (gene set version 1.0[17]), *Bombyx mori* (gene set version 1.0[18]), *Calopteryx splendens* (OGS version 1.0[19]), *Camponotus floridanus* (gene set version 3.3[20]), *Catajapyx aquilonaris* (gene set Caqu_2.0, NCBI GCA_000934665.2[21]), *Centruroides sculpturatus* (OGS version 2.0[22]), *Cimex lectularius* (OGS version 1.2[23]), *Daphnia pulex* (OGS version 1.0[24]), *Dendroctonus ponderosae* (OGS version 1.0[25]), *Drosophila melanogaster* (OGS version r6.03[26]), *Drosophila simulans* (OGS version r1.4[27]), *Eriocheir sinensis* (gene set version 1.0; NCBI GCA_013436485.1[28]), *Eurytemora affinis* (gene set version AFF_2.0; NCBI GCA_000591075.2[29]), *Harpegnathos saltator* (OGS version 3.3[20]), *Hyalella azteca* (gene set version Hazt_2.0[30]), *Ixodes scapularis* (gene set version JCVI_ISG_i3_1.0[31]), *Latrodectus hesperus* (gene set version Lhes_2.0; NCBI GCA_000697925.2), *Lepeophtheirus salmonis* (gene set version LSal-F-Atl-CAN-001; NCBI 001005205.1), *Limulus polyphemus* (gene set version 2.1.2; NCBI GCF_000517525.1[32]), *Macrotermes natalensis* (gene set version 1.2[33]), *Manduca sexta* (OGS version 2[34]), *Nasonia vitripennis* (OGS version 2.26[35]), *Parasteatoda tepidariorum* (gene set version aug3.1[22]), *Pediculus humanus* (gene set version U2.1[36]), *Rhodnius prolixus* (gene set version. C1.2[37]), *Sarcoptes scabiei* (gene set version SscaA1[38]), *Stegodyphus mimosarum* (gene set version 1; NCBI GCA_000611955.2[39]), *Strigamia maritima* (gene set version

Smar1[40]), *Tetranychus urticae* (gene set version 1.0[41]), *Tribolium castaneum* (OGS version Tcas2.0[42]), *Tigriopus californicus* (gene set version Tcal_SD_v2.1; NCBI GCA_007210705.1), *Zootermopsis nevadensis* (OGS version 2.2[43]).

Gene tree inference

The amino acid sequences of a given gene family were aligned with MAFFT[44] version 7.123 and applying the run parameters "--maxiterate 1000 --globalpair –reorder". The resulting multiple amino acid sequence alignments were analyzed with IQ-TREE[45] version 1.6 to infer a gene tree of each gene family under the maximum likelihood optimality criterion. We used the corrected Akaike information criterion (AICc) to select the best-fitting amino acid substitution model for each gene family separately (IQ-TREE option: "-m TESTNEW -msub nuclear -madd LG4M, LG4X"). Branch support was inferred by using 1) ultrafast bootstrapping (UFBoot2) and specifying the run parameters "-bb 10000" and "-bnni" and 2) Shimodaira-Hasegawa approximate likelihood ratio tests ([SH]-aLRT; IQ-TREE option: -alrt 10000)[46]. Branch support was considered reliable if SH-aLRT was larger or equal 80 % and UFboot was larger or equal 95 %. We used FigTree version 1.4.3 (http://tree.bio.ed.ac.uk/software/figtree/) to midway-root the inferred gene trees. Because FigTree is known to map in some situations branch support values to wrong branches when re-rooting a tree[47], we visually verified all bootstrap values with the Interactive Tree of Life (ITOL)[48] version 3 online tool. The gene trees were further processed and annotated, using information from FlyAtlas2[49], with

Inkscape 1.1 (http://www.inkscape.org/). The width of each clade was computed as the number of gene copies per species within each clade.

Modifications applied for whole mount in situ hybridization

Whole-mount RNA *in situ* hybridization was done following the protocol for staining genes in brains and ovaries of adults given by [50] with the following modifications: step 14, probes were diluted in 300 μL (not in 50 μL); step 17, tissues were kept in wash buffer at 57 °C for 1 h (not overnight); step 20, we used the anti-Fluorescein-AP Fab fragment [150 Units] (Roche, Mannheim, Germany) or the anti-Digoxigenin-AP Fab fragment [150 Units] (Roche, Mannheim, Germany) antibodies depending on the riboprobes and on the RNA labelling mix used to synthesize it. Stained samples were rehydrated into PBS, dissected and mounted before imaging.

Gene selection for knockdown experiments

Genes selected for knockdown experiments were found expressed in oenocytes (except *GB51238*, see below), showed a high absolute log_2-fold change value (*Supplementary Tables 7*, *8*, and *9*), and were chosen to represent a variety of gene families (we tried to investigate at least one gene per gene families). We decided to investigate desaturase *GB51238*, even though it was found to be expressed in trophocytes, since it was one of the three co-orthologs of the gene candidate *g14712*. While the other two genes are

expressed in oenocytes, we intentionally decided to select *GB51238* to assess a possible involvement also of trophocytes in CHC biosynthesis.

Double-stranded RNA synthesis

We synthesized dsRNA from the same 387–774-bp-long DNA fragments that we had cloned to facilitate anti-sense riboprobe synthesis. Specifically, template DNA for dsRNA synthesis was generated by PCR-amplifying with the Phusion High Fidelity kit (New England Biolabs, Ipswich, MA, USA) the plasmid inserts using an oligonucleotide primer listed for the specific gene in *Supplementary Table 10* at one end and a T7 oligonucleotide primer at the other end of the plasmid insert. By reversing the order of the oligonucleotides, we obtained dsRNA template DNA with the T7 promotor sequence on different sides of the amplicon.

Reference supplementary Methods

1. Bolger, A. M., Lohse, M. & Usadel, B. Trimmomatic: a flexible trimmer for Illumina sequence data. *Bioinformatics* **30**, 2114–2120 (2014).

2. Petersen, M., Armisén, D., Gibbs, R. A., Hering, L., Khila, A., Mayer, G., Richards, S., Niehuis, O., Misof, B. Diversity and evolution of the transposable element repertoire in arthropods with particular reference to insects. *BMC Evol. Biol.* **19**, 1–15 (2019).

3. Jurka, J., Kapitonov, V. V., Pavlicek, A., Klonowski, P., Kohany, O., Walichiewicz, J. Repbase Update, a database of eukaryotic repetitive elements. *Cytogenet. Genome Res.* **110**, 462–467 (2005). Accessed 1 Sept 2016.

4. Bailly-Bechet M., Haudry A., Lerat E. One code to find them all: a Perl tool to conveniently parse RepeatMasker output files. *Mob. DNA* **5**, 13 (2014).

5. Hoff, K. J., Lange, S., Lomsadze, A., Borodovsky, M. & Stanke, M. BRAKER1: unsupervised RNA-Seq-based genome annotation with GeneMark-ET and AUGUSTUS. *Bioinformatics* **32**, 767–769 (2016).

6. Kim, D., Langmead, B. & Salzberg, S. L. HISAT: a fast spliced aligner with low memory requirements. *Nat. Methods* **12**, 357–360 (2015).

7. Kim, D., Paggi, J.M., Park, C. et al. Graph-based genome alignment and genotyping with HISAT2 and HISAT-genotype. *Nat. Biotechnol.* **37**, 907–915 (2019).

8. Li, H., Handsaker, B., Wysoker, A., Fennell, T., Ruan, J., Homer, N., Marth, G., Abecasis, G. & Durbin, R. The sequence alignment/map format and SAMtools. *Bioinformatics* **25**, 2078–2079 (2009).

9. Barnett, D. W., Garrison, E. K., Quinlan, A. R., Strömberg, M. P. & Marth, G. T. BamTools: a C++ API and toolkit for analyzing and managing BAM files. *Bioinformatics* **27**, 1691–1692 (2011).

10. Besemer, J. & Borodovsky, M. GeneMark: web software for gene finding in prokaryotes, eukaryotes and viruses. *Nucleic acids Res.* **33**, W451–W454 (2005).

11. Stanke, M., Steinkamp, R., Waack, S. & Morgenstern, B. AUGUSTUS: a web server for gene finding in eukaryotes. *Nucleic acids Res.* **32**, W309–W312 (2004).

12. Camacho C., Coulouris G., Avagyan V., Ma N., Papadopoulos J., Bealer K. & Madden T.L. "BLAST+: architecture and applications." *BMC Bioinformatics* **10**, 421 (2008).

13. Johnson, M., Zaretskaya, I., Raytselis, Y., Merezhuk, Y., McGinnis, S. & Madden, T. L. (2008). NCBI BLAST: a better web interface. *Nucleic Acids Res.* **36**, W5–W9 (2008).

14. Nygaard, S., Zhang, G., Schiøtt, M., Li, C., Wurm, Y., Hu, H., et al. The genome of the leaf-cutting ant *Acromyrmex echinatior* suggests key adaptations to advanced social life and fungus farming. *Genome Res.* **21**, 1339–1348 (2011).

15. Nene, V., Wortman, J. R., Lawson, D., Haas, B., Kodira, C., Tu, Z. J., et al. Genome sequence of *Aedes aegypti*, a major arbovirus vector. *Science* **316**, 1718–1723 (2007).

16. Elsik, C. G., Worley, K. C., Bennett, A. K., Beye, M., Camara, F., Childers, C. P., de Graaf, D. C., Debyser, G., Deng, J., Devreese, B., et al. Finding the missing honey bee genes: lessons learned from a genome upgrade. *BMC Genomics* **15**, 1–29 (2014).

17. Sadd, B. M., Barribeau, S. M., Bloch, G., De Graaf, D. C., Dearden, P., Elsik, C. G., et al. The genomes of two key bumblebee species with primitive eusocial organization. *Genome Biol.* **16**, 1–32 (2015).

18. International Silkworm Genome Consortium. The genome of a lepidopteran model insect, the silkworm *Bombyx mori*. *Insect Biochem. Mol. Biol.* **38**, 1036–1045 (2008).

19. Ioannidis, P., Simao, F. A., Waterhouse, R. M., Manni, M., Seppey, M., Robertson, H. M., Misof, B., Niehuis, O., Zdobnov, E. M. Genomic features of the damselfly *Calopteryx splendens* representing a sister clade to most insect orders. *Genome Biol. Evol.* **9**, 415–430 (2017).

20. Bonasio, R., Zhang, G., Ye, C., Mutti, N. S., Fang, X., Qin, N., Donahue, G., Yang, P., Li, Q., Li, C., Zhang, P., Huang, Z., Berger, S. L., Reinberg, D., Wang, J., Liebig, J. Genomic comparison of the ants *Camponotus floridanus* and *Harpegnathos saltator*. *Science* **329**,1068–1071 (2010).

21. Thomas, G. W. C., Dohmen, E., Hughes, D. S. T., Murali, S. C., Poelchau, M., Glastad, K., Anstead, C. A., Ayoub, N. A., Batterham, P., Bellair, M. et al. Gene content evolution in the arthropods. *Genome Biol.* **21**, 15 (2020).

22. Schwager, E. E., Sharma, P. P., Clarke, T., Leite, D. J., Wierschin, T., Pechmann, M., et al. The house spider genome reveals an ancient whole-genome duplication during arachnid evolution. *BMC Biol.* **15**, 1–27 (2017).

23. Rosenfeld, J. A., Reeves, D., Brugler, M. R., Narechania, A., Simon, S., Durrett, R., et al. Genome assembly and geospatial phylogenomics of the bed bug *Cimex lectularius*. *Nat. Commun.* **7**, 1–10 (2016).

24. Colbourne, J. K., Pfrender, M. E, Gilbert, D., Thomas, W. K., Tucker, A., Oakley, T. H., Tokishita, S., Aerts, A., Arnold, G. J., Basu, M. K., et al. The ecoresponsive genome of *Daphnia pulex*. *Science* **331**, 555–561 (2011).

25. Keeling, C. I., Yuen, M. M., Liao, N. Y., Docking, T. R., Chan, S. K., Taylor, G. A., et al. Draft genome of the mountain pine beetle, *Dendroctonus ponderosae* Hopkins, a major forest pest. *Genome Biol.* **14**, 1–20 (2013).

26. Adams, M. D., Celniker, S. E., Holt, R. A., Evans, C. A., Gocayne, J. D., Amanatides, P. G., Scherer, S. E., Li, P.W. et al. The genome sequence of *Drosophila melanogaster*. *Science* **287**, 2185–2195 (2000).

27. Hu, T. T., Eisen, M. B., Thornton, K. R., Andolfatto, P. A second-generation assembly of the *Drosophila simulans* genome provides new insights into patterns of lineage-specific divergence. *Genome Res*. **23**, 89–98 (2013).

28. Cui, Z., Liu, Y., Yuan, J., Zhang, X., Ventura, T., Ma, K. Y., Sun, S., Song, C., Zhan, D., Yang, Y. et al. The Chinese mitten crab genome provides insights into adaptive plasticity and developmental regulation. *Nat. Commun*. **12**, 2395 (2021).

29. Choi, B. S., Kim, D. H., Kim, M. S., Park, J. C., Lee, Y. H., Kim, H. J., Jeong, C. B., Hagiwara, A., Souissi, S., Lee, J. S. The genome of the European estuarine calanoid copepod *Eurytemora affinis*: Potential use in molecular ecotoxicology. *Mar. Pollut. Bull.* **166**, 112190 (2021).

30. Poynton, H. C., Hasenbein, S., Benoit, J. B., Sepulveda, M. S., Poelchau, M. F., Hughes, D. S., et al. The toxicogenome of *Hyalella azteca*: a model for sediment ecotoxicology and evolutionary toxicology. *Environ. Sci. Technol.* **52**, 6009–6022 (2018).

31. Gulia-Nuss, M., Nuss, A. B., Meyer, J. M., Sonenshine, D. E., Roe, R. M., Waterhouse, R. M., et al. Genomic insights into the *Ixodes scapularis* tick vector of Lyme disease. *Nat. Commun.* **7**, 1–13 (2016).

32. Simpson, S. D., Ramsdell, J. S., Watson III, W. H. & Chabot, C. C. (2017). The draft genome and transcriptome of the Atlantic horseshoe crab, *Limulus polyphemus*. *Int. J. Genomics* **2017**, 7636513 (2017).

33. Poulsen, M., Hu, H., Li, C., Chen, Z., Xu, L., Otani, S., et al. Complementary symbiont contributions to plant decomposition in a fungus-farming termite. *Proc. Natl. Acd. Sci. USA* **111**, 14500–14505 (2014).

34. Kanost, M. R., Arrese, E. L., Cao, X., Chen, Y. R., Chellapilla, S., Goldsmith, M. R., et al. Multifaceted biological insights from a draft genome sequence of the tobacco hornworm moth, *Manduca sexta*. *Insect Biochem. Mol. Biol.* **76**, 118–147 (2016).

35. Werren, J. H., Richards, S., Desjardins, C. A., Niehuis, O., Gadau, J., Colbourne, J. K. et al. Functional and evolutionary insights from the genomes of three parasitoid Nasonia species. *Science* **327**, 343–348 (2010).

36. Kirkness, E. F., Haas, B. J., Sun, W., Braig, H. R., Perotti, M. A., Clark, J. M., et al. Genome sequences of the human body louse and its primary endosymbiont provide insights into the permanent parasitic lifestyle. *Proc. Natl. Acad. Sci. USA* **107**, 12168–12173 (2010).

37. Mesquita, R. D., Vionette-Amaral, R. J., Lowenberger, C., Rivera-Pomar, R., Monteiro, F. A., Minx, P., et al. Genome of *Rhodnius prolixus*, an insect vector of Chagas disease, reveals unique adaptations to hematophagy and parasite infection. *Proc. Natl. Acad. Sci. USA* **112**, 14936–14941 (2015).

38. Rider, S. D., Morgan, M. S. & Arlian, L. G. Draft genome of the scabies mite. *Parasit. Vectors* **8**, 1–14 (2015).

39. Sanggaard, K. W., Bechsgaard, J. S., Fang, X., Duan, J., Dyrlund, T. F., Gupta, V., et al. Spider genomes provide insight into composition and evolution of venom and silk. *Nat. Commun.* **5**, 1–12 (2014).

40. Chipman, A. D., Ferrier, D. E., Brena, C., Qu, J., Hughes, D. S., Schröder, R., Torres-Oliva, M., Znassi, N., Jiang, H., Almeida, F. C., Alonso, C. R., Apostolou, Z. et al. The first myriapod genome sequence reveals conservative arthropod gene content and

genome organisation in the centipede *Strigamia maritima*. *PLoS Biol.* **12**, e1002005 (2014).

41. Grbić, M., Van Leeuwen, T., Clark, R. M., Rombauts, S., Rouzé, P., Grbić, V., et al. The genome of *Tetranychus urticae* reveals herbivorous pest adaptations. *Nature* **479**, 487–492 (2011).

42. Tribolium Genome Sequencing Consortium. The genome of the model beetle and pest *Tribolium castaneum*. *Nature* **452**, 949–955 (2008).

43. Terrapon, N., Li, C., Robertson, H. M., Ji, L., Meng, X., Booth, W., Chen, Z., Childers, C. P., Glastad, K. M., Gokhale, K., Gowin, J., Gronenberg, W. et al. Molecular traces of alternative social organization in a termite genome. *Nat. Commun*. **5**, 3636 (2014).

44. Katoh, K. & Standley, D. M. MAFFT multiple sequence alignment software version 7: improvements in performance and usability. *Mol. Biol. Evol.* **30**, 772–780 (2013).

45. Nguyen, L. T., Schmidt, H. A., Von Haeseler, A. & Minh, B. Q. IQ-TREE: a fast and effective stochastic algorithm for estimating maximum-likelihood phylogenies. *Mol. Biol. Evol.* **32**, 268–274 (2015).

46. Guindon, S., Dufayard, J. F., Lefort, V., Anisimova, M., Hordijk, W. & Gascuel, O. New algorithms and methods to estimate maximum-likelihood phylogenies: assessing the performance of PhyML 3.0. *Syst. Biol.* **59**, 307–321 (2010).

47. Czech, L., Huerta-Cepas, J. & Stamatakis, A. A critical review on the use of support values in tree viewers and bioinformatics toolkits. *Mol. Biol. Evol.* **34**, 1535–1542 (2017).

48. Letunic, I. & Bork, P. Interactive tree of life (iTOL) v3: an online tool for the display and annotation of phylogenetic and other trees. *Nucleic Acids Res.* **44**, W242–W245 (2016).

49. Leader, D. P., Krause, S. A., Pandit, A., Davies, S. A. & Dow, J. A. T. FlyAtlas 2: a new version of the Drosophila melanogaster expression atlas with RNA-Seq, miRNA-Seq and sex-specific data. *Nucleic Acids Res.* **46**, D809–D815 (2018).

50. Dearden, P. K., Duncan, E. J. & Wilson, M. J. Whole-mount in situ hybridization of honeybee (*Apis mellifera*) tissues. *Cold Spring Harb. Protoc.* **2009**, pdb-prot5225 (2009).

Supplementary Tables

Supplementary Table 1. Sample dates of *Odynerus spinipes* females, kept in the laboratory and whose cuticular hydrocarbon profile (chemotype) we studied multiple times over the course of their adult life.

Sample ID	Chemo-type	Eclosion date	1st sampling date	Age at 1st sampling date	2nd sampling date	Age at 2nd sampling date	3rd sampling date	Age at 3rd sampling date
ON_8469[a]	1	April 25, 2016	April 26, 2016	1	May 4, 2016	9	May 11, 2016	16
ON_8471[b]	2	April 25, 2016	April 26, 2016	1	May 2, 2016	7	May 9, 2016	14
ON_8473	2	April 25, 2016	April 27, 2016	2	May 2, 2016	7	May 10, 2016	15
ON_8475[c]	1	April 25, 2016	April 26, 2016	1	May 2, 2016	7	NA	NA
ON_8477[d]	2	April 25, 2016	April 26, 2016	1	May 4, 2016	9	NA	NA
ON_8479	1	April 25, 2016	April 27, 2016	2	May 2, 2016	7	May 10, 2016	15
ON_8481	1	April 25, 2016	April 27, 2016	2	May 2, 2016	7	May 10, 2016	15
ON_8483	1	April 25, 2016	April 27, 2016	2	May 2, 2016	7	May 10, 2016	15
ON_8485	2	April 25, 2016	April 27, 2016	2	May 3, 2016	8	May 9, 2016	14
ON_8487	1	April 25, 2016	April 27, 2016	2	May 2, 2016	7	NA	NA
ON_8489	2	April 25, 2016	April 27, 2016	2	May 3, 2016	8	May 10, 2016	15
ON_8491	2	April 26, 2016	April 27, 2016	1	May 3, 2016	7	May 9, 2016	13
ON_8493	2	April 26, 2016	April 28, 2016	2	May 3, 2016	7	May 10, 2016	14
ON_8495	2	April 26, 2016	April 28, 2016	2	May 2, 2016	6	NA	NA
ON_8497	2	April 26, 2016	April 28, 2016	2	May 2, 2016	6	NA	NA
ON_8499	1	April 26, 2016	April 28, 2016	2	May 3, 2016	7	May 9, 2016	13
ON_8501	2	April 26, 2016	April 28, 2016	2	May 3, 2016	7	May 9, 2016	13
ON_8503	2	April 26, 2016	April 28, 2016	2	May 3, 2016	7	May 10, 2016	14
ON_8505	2	April 26, 2016	April 28, 2016	2	May 3, 2016	7	May 9, 2016	13
ON_8507	2	April 27, 2016	April 29, 2016	2	May 4, 2016	7	May 10, 2016	13
ON_8509	1	April 27, 2016	April 29, 2016	2	May 2, 2016	5	NA	NA
ON_8511	2	April 27, 2016	April 29, 2016	2	May 4, 2016	7	May 10, 2016	13
ON_8513	1	April 27, 2016	April 29, 2016	2	May 4, 2016	7	May 11, 2016	14
ON_8515	2	April 27, 2016	April 29, 2016	2	May 4, 2016	7	May 11, 2016	14
ON_8517	2	April 27, 2016	April 29, 2016	2	May 4, 2016	7	May 11, 2016	14
ON_8519	1	April 27, 2016	April 28, 2016	1	May 4, 2016	7	May 11, 2016	14
ON_8521	1	April 28, 2016	April 29, 2016	1	NA	NA	NA	NA
ON_8523	2	April 27, 2016	April 29, 2016	2	May 4, 2016	7	May 11, 2016	14
ON_8525	2	April 28, 2016	April 29, 2016	1	NA	NA	NA	NA
ON_8527	1	April 28, 2016	April 29, 2016	1	NA	NA	NA	NA

[a] Extracurricular sampling at April 29, 2016 (wasp's age: 4 days)
[b] Extracurricular sampling at April 27, 2016 (wasp's age: 3 days)
[c] Extracurricular sampling at April 27, 2016 (wasp's age: 2 days)
[c] Extracurricular sampling at April 29, 2016 (wasp's age: 4 days)

Supplementary Table 2. Sample dates of *Odynerus spinipes* females whose cuticular hydrocarbon profile (chemotype) we studied multiple times over the course of their adult life at two field sites (Büchelberg [B; 49.027985, 8.164801] and Tenneville [T; 50.100671, 5.530061]).

Sample ID	Chemo-type	1st sampling date	Idle time (days)	2nd sampling date	Idle time (days)	3rd sampling date	Idle time (days)	4th sampling date	Field site
ON_8651	1	June 7, 2016	8	June 15, 2016	NA	NA	NA	NA	B
ON_8653	1	June 7, 2016	13	June 20, 2016	NA	NA	NA	NA	B
ON_8663	1	June 15, 2016	5	June 20, 2016	NA	NA	NA	NA	B
ON_8671	2	May 27, 2017	4	Mai 31, 2017	8	June 8, 2017	NA	NA	B
ON_8673	1	May 27, 2017	6	June 2, 2017	6	June 8, 2017	11	June 19, 2017	B
ON_8675	1	May 31, 2017	8	June 8, 2017	11	June 19, 2017	NA	NA	B
ON_8677	2	May 31, 2017	8	June 8, 2017	NA	NA	NA	NA	B
ON_8693	1	June 10, 2017	3	June 13, 2017	2	June 15, 2017	NA	NA	B
ON_8699	2	June 10, 2017	3	June 13, 2017	NA	NA	NA	NA	B
ON_8709	2	June 13, 2017	6	June 19, 2017	3	June 21, 2017	NA	NA	B
ON_8721	2	June 21, 2017	2	June 23, 2017	NA	NA	NA	NA	B
ON_8723	2	June 21, 2017	2	June 23, 2017	NA	NA	NA	NA	B
ON_8725	1	July 3, 2017	2	July 5, 2017	NA	NA	NA	NA	T
ON_8729	2	July 3, 2017	2	July 5, 2017	NA	NA	NA	NA	T
ON_8733	2	July 3, 2017	3	July 6, 2017	NA	NA	NA	NA	T
ON_8735	2	July 3, 2017	3	July 6, 2017	NA	NA	NA	NA	T
ON_8737	2	July 4, 2017	2	July 6, 2017	NA	NA	NA	NA	T
ON_8745	1	July 4, 2017	2	July 6, 2017	NA	NA	NA	NA	T

Supplementary Table 3. Information to *Odynerus spinipes* transcriptomes we sequenced and used to facilitate annotation of the *Odynerus spinipes* draft genome.

Sample ID	Tissue ID	Sex	Chemo-type	Tissue	Sampling location	Collection date	Genbank acc. no.
ON_6860	Odsp_RNA_05	female	1	whole body	Büchelberg	May 19, 2014	SRR14729246
ON_6859	Odsp_RNA_09	female	2	whole body	Büchelberg	May 19, 2014	SRR14729245
ON_6846	Odsp_RNA_08	male	NA	whole body	Büchelberg	May 18, 2014	SRR14729244

Supplementary Table 4. Adult *Odynerus spinipes* females whose metasoma transcriptomes we sequenced and which we collected near Bad Muskau (51.548200, 14.714900), Büchelberg (49.027985, 8.164801), and Eichenzell (50.495927, 9.704589).

Sample ID	GenBank accession number	Chemo-type	Collection site	RNA fixation date	Age class	Batch no.
ON_7724	SAMN14256292	1	Büchelberg	January 27, 2015	12–38 h	1
ON_7728	SAMN14256293	1	Büchelberg	January 29, 2015	12–38 h	1
ON_7747	SAMN14256294	1	Büchelberg	February 12, 2015	12–38 h	1
ON_7723	SAMN14256295	2	Büchelberg	January 27, 2015	12–38 h	1
ON_7730	SAMN14256296	2	Büchelberg	January 29, 2015	12–38 h	1
ON_7739	SAMN14256297	2	Büchelberg	January 31, 2015	12–38 h	1
ON_8683	SAMN14256301	1	Büchelberg	June 3, 2017	48–62 h	2
ON_8685	SAMN14256302	1	Büchelberg	June 3, 2017	48–62 h	2
ON_12769	SAMN14256298	1	Eichenzell	June 12, 2016	48–62 h	2
ON_8687	SAMN14256303	2	Büchelberg	June 6, 2017	48–62 h	2
ON_12771	SAMN14256299	2	Eichenzell	June 14, 2016	48–62 h	2
ON_12775	SAMN14256300	2	Bad Muskau	May 17, 2017	48–62 h	2

Supplementary Table 5. *Odynerus spinipes* genes significantly differentially expressed in batch 1-females with different chemotype. The table shows the gene IDs, the log_2-fold change values (values < 0 indicate higher expression of genes in females with chemotype 2 than in females with chemotype 1), the *p*-value obtained with DESeq2, the adjusted *p*-value from applying the false discovery rate (FDR), and the predicted function of the genes based on homology.

Gene	Mean	Log2fold-	pval	FDR	Predicted function based on homology
g10050	3237.95	5.92	0.000	0.000	elongation of very long chain fatty acids protein 1-like
g11859	808.04	5.08	0.000	0.000	NA
g10048	3800.63	4.81	0.000	0.000	elongation of very long chain fatty acids protein 1-like
g10049	2635.75	3.83	0.000	0.000	elongation of very long chain fatty acids protein 1-like
g7007	1083.91	3.44	0.000	0.000	elongation of very long chain fatty acids protein 1-like
g5191	250.68	1.83	0.000	0.000	venom protein
g14437	38.71	1.68	0.000	0.000	endonuclease-reverse transcriptase
g9972	21.72	1.29	0.000	0.000	histone-lysine N-methyltransferase SETMAR-like
g9971	36.39	1.34	0.000	0.000	PREDICTED: histone-lysine N-methyltransferase SETMAR-like
g12443	5803.23	1.20	0.000	0.000	elongation of very long chain fatty acids protein 1-like
g3041	1484.29	1.13	0.000	0.000	serine-rich adhesin for platelets
g13710	100.86	1.09	0.000	0.000	mucin-3A
g14860	120.01	1.20	0.000	0.001	NA
g8981	1988.04	1.11	0.000	0.003	glycosyltransferase family 1 protein
g2126	332.91	1.15	0.000	0.003	arylphorin subunit alpha-like
g6203	1174.03	1.14	0.000	0.003	tetra-peptide repeat homeobox protein 1-like
g13132	1732.04	0.74	0.000	0.010	transient receptor potential cation channel trpm
g3744	142162.00	0.84	0.000	0.010	apolipophorins
g7125	1737.39	0.66	0.000	0.011	lipoamide acyltransferase component of branched-chain alpha-keto acid dehy-
g7239	107.52	0.96	0.000	0.011	Protein apterous
g4311	1176.17	0.82	0.000	0.016	DEAD-box ATP-dependent RNA helicase 20-like
g785	16782.44	0.87	0.000	0.024	pupal cuticle protein-like
g11772	175.40	0.96	0.000	0.025	diacylglycerol kinase kappa
g8320	27.63	0.96	0.000	0.029	PREDICTED: piggyBac transposable element-derived protein 4-like
g8919	67.30	0.93	0.000	0.036	NA
g3244	2434.25	0.70	0.000	0.046	formin-2-like
g3764	634.11	0.95	0.000	0.049	cytochrome P450 4g15-like
g12825	729.30	0.75	0.000	0.049	adenylate kinase isoenzyme 5
g3930	10.29	0.81	0.000	0.049	NA
g501	626.61	0.72	0.000	0.049	farnesol dehydrogenase-like
g10135	279.53	-4.76	0.000	0.000	Retrovirus-related Pol polyprotein from transposon TNT 1-94
g14547	58.50	-2.54	0.000	0.000	retrovirus-related pol polyprotein from transposon tnt 1-94
g7008	6557.42	-2.09	0.000	0.000	elongation of very long chain fatty acids protein 1-like
g3158	773.28	-1.79	0.000	0.000	fatty acid synthase-like
g3059	2296.06	-1.55	0.000	0.000	secretin receptor-like
g7616	10013.21	-1.19	0.000	0.000	elongation of very long chain fatty acids protein AAEL008004-like
g8887	24.51	-1.31	0.000	0.000	gag-pol polyprotein
g11719	158.60	-1.28	0.000	0.000	Copia protein
g1571	917.36	-1.27	0.000	0.000	putative fatty acyl-CoA reductase CG5065
g2403	3821.09	-0.67	0.000	0.000	mitochondrial cardiolipin hydrolase-like protein
g283	420.58	-0.96	0.000	0.000	rhythmically expressed gene 5 protein
g6414	346.83	-1.04	0.000	0.003	alanine--glyoxylate aminotransferase 2 homolog 1. mitochondrial
g1635	8335.84	-1.08	0.000	0.006	group XV phospholipase A2-like
g13265	1344.43	-1.10	0.000	0.007	fatty acid-binding protein, adipocyte-like
g14712	26410.19	-0.99	0.000	0.008	PREDICTED: acyl-CoA Delta(11) desaturase-like
g11240	21.69	-0.96	0.000	0.013	transposable element tcb1 transposase
g13220	227.21	-1.02	0.000	0.014	PREDICTED: odorant receptor 47a-like
g1176	774.11	-0.72	0.000	0.018	octopamine receptor beta-3R-like
g3272	58.24	-1.02	0.000	0.020	ranBP-type and C3HC4-type zinc finger-containing protein 1-like
g2861	593.75	-0.67	0.000	0.024	NA
g7590	672.52	-0.72	0.000	0.027	ADP-ribosylation factor-like protein 13B
g2290	1358.50	-0.84	0.000	0.029	fatty-acid amide hydrolase 2-B-like
g4379	88.95	-0.92	0.000	0.039	cytochrome b5-like
g5819	151.70	-0.89	0.000	0.040	enhancer of split mgamma protein-like
g14091	153.94	-0.89	0.000	0.043	gustatory receptor 5a for trehalose-like
g11201	27.79	-0.93	0.000	0.043	zinc finger DNA binding protein
g4709	917.27	-0.91	0.000	0.043	alpha-tocopherol transfer protein-like
g8089	55.04	-0.93	0.000	0.043	rna-dependent dna polymerase
g2711	274.01	-0.92	0.000	0.046	interaptin-like

g9118	248.86	-0.89	0.000	0.047	atp-binding protein

Supplementary Table 6. *Odynerus spinipes* genes significantly differentially expressed in batch 2-females with different chemotype. The table shows the gene IDs, the log_2-fold change values (values < 0 indicate higher expression of genes in females with chemotype 2 than in females with chemotype 1), the *p*-value obtained with DESeq2, the adjusted *p*-value from applying the false discovery rate (FDR), and the predicted function of the genes based on homology.

Gene id	Mean	Log2fold-change	*p*	FDR	Predicted function based on homology
g1053	31045.54	1.68	0.000	0.004	maternal effect protein oskar
g7363	627.12	1.69	0.000	0.007	probable phospholipid-transporting ATPase IF
g7362	87.64	2.27	0.000	0.008	2-oxoglutarate dehydrogenase, mitochondrial-like
g13468	4344.05	2,17	0.000	0.013	ejaculatory bulb-specific protein 3-like
g3754	615.06	1.90	0.000	0.013	lipase 3-like
g5503	59.11	2.14	0.000	0.013	broad-complex core protein isoforms 1/2/3/4/5-like
g499	172.13	1.74	0.000	0.030	NA
g13957	754.88	2.01	0.000	0.032	synaptic vesicle glycoprotein 2C-like
g7599	891.43	1.92	0.000	0.032	NA
g5507	357.76	1.97	0.000	0.036	broad-complex core protein isoforms 1/2/3/4/5-like
g7908	1069.05	1.97	0.000	0.036	follicle cell protein 3C-1
g9268	178.33	1.93	0.000	0.0419	location of vulva defective 1-like
g4638	1877.10	1.09	0.000	0.0493	lethal(2) giant larvae protein homolog 1
g14712	36159.52	-3.85	0.000	0.000	acyl-CoA Delta(11) desaturase-like
g283	2091.25	-1.73	0.000	0.004	rhythmically expressed gene 5 protein
g14676	2532.97	-1.75	0.000	0.008	1-acyl-sn-glycerol-3-phosphate acyltransferase beta
g3036	397.44	-2.07	0.000	0.008	heat shock protein 70 A2-like
g6760	340.61	-2.25	0.000	0.008	tektin-B1
g3696	116.54	-2.11	0.000	0.0128	allatostatins
g531	2218.22	-1.70	0.000	0.0128	progestin and adipoQ receptor family member 3
g1906	83.61	-1.91	0.000	0.0128	sodium-dependent nutrient amino acid transporter 1-like
g1903	87.59	-1.64	0.000	0.0161	intraflagellar transport protein 46 homolog
g804	8223.24	-1.67	0.000	0.0241	long-chain fatty acid transport protein 4-like
g5799	117.19	-1.38	0.000	0.030	CD63 antigen
g2073	10007.80	-2.03	0.000	0.030	defensin-1-like
g10320	7903.07	-1.65	0.000	0.032	acyl-CoA-binding protein homolog
g3695	4411.92	-1.41	0.000	0.043	protein amnionless-like
g12587	602.56	-1.29	0.000	0.048	tyrosine-protein kinase transmembrane receptor Ror-like
g12803	6816.84	-1.41	0.000	0.048	organic cation transporter protein-like
g3526	15054.44	-1.92	0.000	0.048	leucine-rich repeat-containing protein 15-like
g7616	29611.98	-1.46	0.000	0.049	elongation of very long chain fatty acids protein AAEL008004-like

Supplementary Table 7. *Odynerus spinipes* genes significantly differentially expressed in females with different chemotype in two batches of samples differing in age and sampling location from each other (***Supplementary Table 4***). The table shows the results from first globally assessing gene expression differences in batch 1 and subsequently assessing whether those genes that are considered differentially expressed based on false discovery rate (FDR) are also differentially expressed (*p*-value ≤ 0.05) in batch 2 after applying Holm-Bonferroni correction for multiple testing (number of genes tested in batch 2) on the *p*-values provided by DESeq2. Log_2-fold change values > 0 indicate higher expression of genes in females with chemotype 1 than in females with chemotype 2. Log_2-fold change values < 0 indicate higher expression of genes in females with chemotype 2 than in females with chemotype 1. Genes in bold were significantly differently expressed in both batches.

Gene ID	Batch 1			Batch 2			Predicted function
	Log_2-fold change	*p*	*p* (adj.)[1]	Log_2-fold change	*p*	*p* (adj.)[2]	
**g283**	-0.96	0.000	0.003	-1.73	0.000	**0.000**	**rhythmically expressed gene 5 protein**
g1571	-1.27	0.000	0.000	-1.09	0.031	1.000	fatty acid reductase
**g2290**	-0.84	0.000	0.029	-1.79	0.000	**0.019**	**fatty acid amide hydrolase**
g3041	1.13	0.000	0.000	-0.89	0.032	1.000	serine-rich adhesin for platelets
**g3059**	-1.55	0.000	0.000	-1.71	0.000	**0.013**	**parathyroid hormone/related receptor**
g3158	-1.79	0.000	0.000	-1.00	0.024	1.000	fatty acid synthase
g4709	-0.91	0.000	0.043	-1.14	0.021	1.000	alpha-tocopherol transfer protein-like
g6414	-1.04	0.000	0.003	-1.19	0.018	0.968	alanine--glyoxylate aminotransferase 2 homolog 1, mitochondrial
**g7616**	-1.19	0.000	0.000	-1.46	0.000	**0.008**	**fatty acid elongase**
g12825	0.75	0.000	0.049	1.01	0.002	0.090	adenylate kinase isoenzyme 5
g14547	-2.54	0.000	0.000	-1.09	0.018	0.968	retrovirus-related pol polyprotein from transposon tnt 1-94
**g14712**	-0.99	0.000	0.008	-3.85	0.000	**0.000**	**fatty acid desaturase**

[1] FDR
[2] Holm-Bonferroni correction for 60 tests

Supplementary Table 8. *Odynerus spinipes* genes significantly differentially expressed in females with different chemotype in two batches of samples differing in age and sampling location from each other (***Supplementary Table 4***). The table shows the results from first globally assessing gene expression differences in batch 2 and subsequently assessing whether those genes that are considered differentially expressed based on false discovery rate (FDR) are also differentially expressed (*p*-value ≤ 0.05) in batch 1 when applying Holm-Bonferroni correction for multiple testing (number of genes tested in batch 1) on the *p*-values provided by DESeq2. Log_2-fold change values < 0 indicate higher expression of genes in females with chemotype 2 than in females with chemotype 1. Genes in bold were significantly differently expressed in both batches.

Gene ID	Batch 2			Batch 1			Predicted function
	Log_2-fold change	*p*	*p* (adj.)[1]	Log_2-fold change	*p*	*p* (adj.)[2]	
**g283**	-1.73	0.000	0.004	-0.96	0.000	**0.000**	**rhythmically expressed gene 5 protein**
g531	-1.70	0.000	0.013	-0.49	0.025	0.685	progestin and adipoQ receptor family member 3 isoform X1
g7599	1.92	0.000	0.032	-0.51	0.048	1.000	NA
**g7616**	-1.46	0.000	0.049	-1.19	0.000	**0.000**	**fatty acid elongase**
g14676	-1.75	0.000	0.008	-0.52	0.024	0.665	1-acyl-sn-glycerol-3-phosphate acyltransferase alpha
**g14712**	-3.85	0.000	0.000	-0.99	0.000	**0.000**	**fatty acid desaturase**

[1] FDR
[2] Holm-Bonferroni correction for 31 tests

Supplementary Table 9. *Odynerus spinipes* genes encoding fatty acid synthases, fatty acid elongases, fatty acid desaturases, and fatty acid reductases and found significantly differentially expressed in females belonging to different age classes (***Supplementary Table 4***). Shown are the results from analyzing the twelve transcriptomes with the two software packages DESeq2 and EdgeR. *Log*$_2$-fold change values > 0 indicate higher expression of genes in 48–62-h-old females than in 12–38-h-old females. *Log*$_2$-fold change values < 0 indicates higher expression of genes in 12–38-h-old females than in 48–62-h-old females. FDR: false discovery rate.

**Gene ID**	**DESeq2**			**EdgeR**			**Gene family**
	***log*$_2$-fold change**	***p***	**FDR**	***log*$_2$-fold change**	***p***	**FDR**	
g1571	3.170	0.001	**0.001**	4.691	0.000	**0.000**	fatty acid reductase
g2413	-0.767	0.003	**0.003**	-0.813	0.002	**0.010**	fatty acid reductase
g3158	1.972	0.017	**0.017**	4.361	0.003	**0.012**	fatty acid synthase
g6537	1.482	0.014	**0.014**	1.739	0.006	**0.022**	fatty acid elongase
g7609	NA	NA	NA	2.711	0.001	**0.004**	fatty acid elongase
g7610	NA	NA	NA	7.095	0.000	**0.000**	fatty acid elongase
g7616	1.454	0.004	**0.004**	1.602	0.002	**0.009**	fatty acid elongase
g7620	1.449	0.036	**0.036**	NA	NA	NA	fatty acid elongase
g7621	NA	NA	NA	3.535	0.001	**0.005**	fatty acid elongase
g7842	1.208	0.004	**0.004**	1.288	0.001	**0.005**	fatty acid reductase
g14708	-0.691	0.013	**0.013**	-0.730	0.004	**0.016**	fatty acid desaturase

Supplementary Table 10. Number of samples from which cuticular hydrocarbon and gene expression data were collected in RNAi experiments. The time between dsRNA injection and data collection is specified in the column with the header **Δ***t.*

Honey bee gene ID	*O. spinipes* gene ID	Predicted function	Δ*t* [days]	Treatment group (RT-qPCR) sample size	Control group (RT-qPCR) sample size
GB40659	g14708	fatty acid desaturase	2	7 (NA)	6 (NA)
GB42218	g14712_18	fatty acid desaturase	4	12 (12)	13 (11)
GB48195	g14710	fatty acid desaturase	3	11 (8)	8 (7)
GB51238	g14712_38	fatty acid desaturase	4	6 (6)	8 (7)
GB51247	g7616	fatty acid elongase	5	10 (8)	6 (6)
GB51250	g7615	fatty acid elongase	3	7 (7)	8 (7)
GB53695	g2290	fatty amid hydrolase	4-5*	10-7* (9)	6 (6)
GB54397	g7610	fatty acid elongase	2	8 (NA)	9 (NA)
GB54404	g7617	fatty acid elongase	4	7 (7)	11 (8)

* We used both samples collected 4 days (10 bees) and 5 days (7 bees) after injection.

Supplementary Table 11. Oligonucleotide primers used to amplify target gene nucleotide stretches in the honey bee, required for *in situ* hybridization probe design and dsRNA synthesis*.*

**Honey beegene ID**	***O. spinipes* gene ID**	**Forward oligonucleotide primer (5’ → 3’)**	**T_m (°C)**	**Reverse oligonucleotide primer (5’ → 3’)**	**T_m (°C)**
GB40659	g14708	CGACTGTGGGCGCATAAAAG	60	TCACCCTCCTGTTGTACGGT	60
GB40681	g7008	TGGTCCAGTGAAGATAGCTCATAC	64	TTCCCATCGTATTCCCAACTGT	60
GB42218	g14712_1	CTCCGAAAGCAAAGAGGGAC	60	ACTGACGCAGATCCGCATAAC	61
GB44756	g283	GTGACGCAAAAGGACTTATTTGG	61	CTTACGCTTTACAATTACCTCTTGA	61
GB46038	g6537	CTCGCTGATGAACGAACGAGA	61	CAATGCGGCCAAACCGTAAT	58
GB48195	g14710	TGGCATTTCAAGATGCAGCAA	57	CCTTATCACCCCAACCCCAC	63
GB49380	g7842	GTTAGATGTGCCGCTGTTCG	60	TGCTGCCAGGTGATTGGATT	60
GB50627	g2413	TGGTCTCGGTTTGTCTTCCG	60	TGTAGATTTCCGACGATCTGCT	59
GB51236	g14712_2	TCTTCAAACAACCGCTTTCCAA	58	ATGCCATCCCTCACCACATC	60
GB51238	g14712_3	ATTATGCCATCCCTCGCCGT	60	TGCGGATCCTCATAATTCCCG	61
GC51247	g7616	ACGAGTACTTGATGAGTGGCT	59	AGCGAGCTTTTTCTCCGTGT	58
GB51250	g7615	TCACACCAGGCGGTCATTC	59	GCATGATCGGGTCACTCGG	62
GB52087	g1571	AAGCAGAGAATGTAGCTGAAGT	58	CGAAGACCTCTCACTCCTTGA	61
GB52590	g3158	CGAAAAGTTACAAGCGAACGGT	60	CTCGATGCACTTGACACCCT	60
GB52820	g3059	AGGAAATTAAGATGTCCCCGGAA	61	ACAGCCACAAAGAAACCTTGG	59
GB53412	g907	CGGTTTTTCAGGACGTTATCCT	60	CGTACACTCTGCGTGCATCT	60
GB53695	g2290	GGTCAACTCTTAACATCCCCAGT	63	TGTAGGCCACCAACAATCTGTAT	61
GB54302	g7621	GCGCTCAGGGTCGCTAGA	61	TGTCTTTGCTATCTCGGTCTCC	62
GB54396	g7611	AAAGGCCATCCAATAACGAAAGA	59	CCGATCGAAGCCAGCATGTA	61
GB54397	g7610	TCCTCGCGCAATAGAGATAACC	62	CGCCAAACAATGATGGACGG	60
GB54399	g7609	ACCTGGATTCAACTGGTGCAA	59	GCCACTTGACGAACCTCCTT	60
GB54401	g7620	GTCGGTGAACAGAAATGGCA	59	TTCCGGTCCCATAGCGGATA	60
GB54404	g7617	CGTTAAGTTCACTCCTGGTGG	61	CGGTTCCTAAGCCCACTGTC	63
GB55040	g858	TTAGACAGTAGGTTGCCGCC	60	CCACATACCACCTGGGACAA	59
GFP	GFP	GGAGAAGAACTTTTCACTGG	59	ATTCTTTTGTTTGTCTGCC	58

Supplementary Table 12. Oligonucleotide primer sequences used to quantify the expression of target genes via quantitative reverse-transcription real-time PCR in RNAi experiments.

**Honey bee gene ID**	**Forward oligonucleotide primer**	**T_m (°C)**	**Forward oligonucleotide primer**	**T_m (°C)**
GB40659	CCGGCCAGGTTACGGATAAT	60	TCCTGTGGTCCCGTATCCAT	60
GB42218	ATGGGATTTGCTGGTTCGAT	56	CTCTGTGGTCCCTTACCCAT	60
GB48195	GCCGCTGTGTATGGATTGTATC	62	GCTGTAATTCCCAATCCAGTGC	62
GB49975	TGCAATGTTGACAGGTTGGT	56	CTCTTGTCCCTTTTCTAGCTGC	62
GB51238	GGATTAACGGAACGAAGGCA	58	TGTGATTCTGGTAAGAGGTTGTT	59
GB51247	AAATCAGATCCCAGAGTGAACCA	61	ACGTGGAGAATAGCGTTTGGA	59
GB51250	CAAATGGTGTTCTCAGCGTGG	61	ACAAAGAAGATCGTGTCCATGAAT	60
GB53695	GGCTATCCCTCTAGCAACCAC	63	ACCACCAGAAGATCCACCAA	58
GB54397	CGATCCTCGCGCAATAGAGAT	61	CGGAGGACAAAGAAGACCGTA	61
GB54404	TAGCAGGATGGGGAGGTCAA	60	ACCACCAACAACCTTCTGCC	60

Supplementary Figures

***Supplementary Figure 1*. Differences in the relative abundance of specific alkenes and of alkadienes in cuticular hydrocarbon profiles of *Odynerus spinipes* females.**

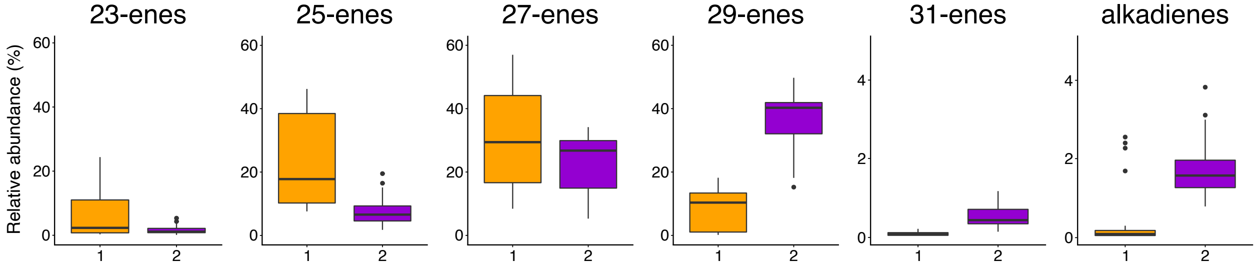

Box plots of the relative abundances of specific alkenes and of alkadienes in females expressing either chemotype 1 (orange) or chemotype 2 (purple).

***Supplementary Figure 2*. Gene tree of fatty acid synthases from 37 species of Euarthropoda showing copy numbers and involvement of genes in CHC biosynthesis.**

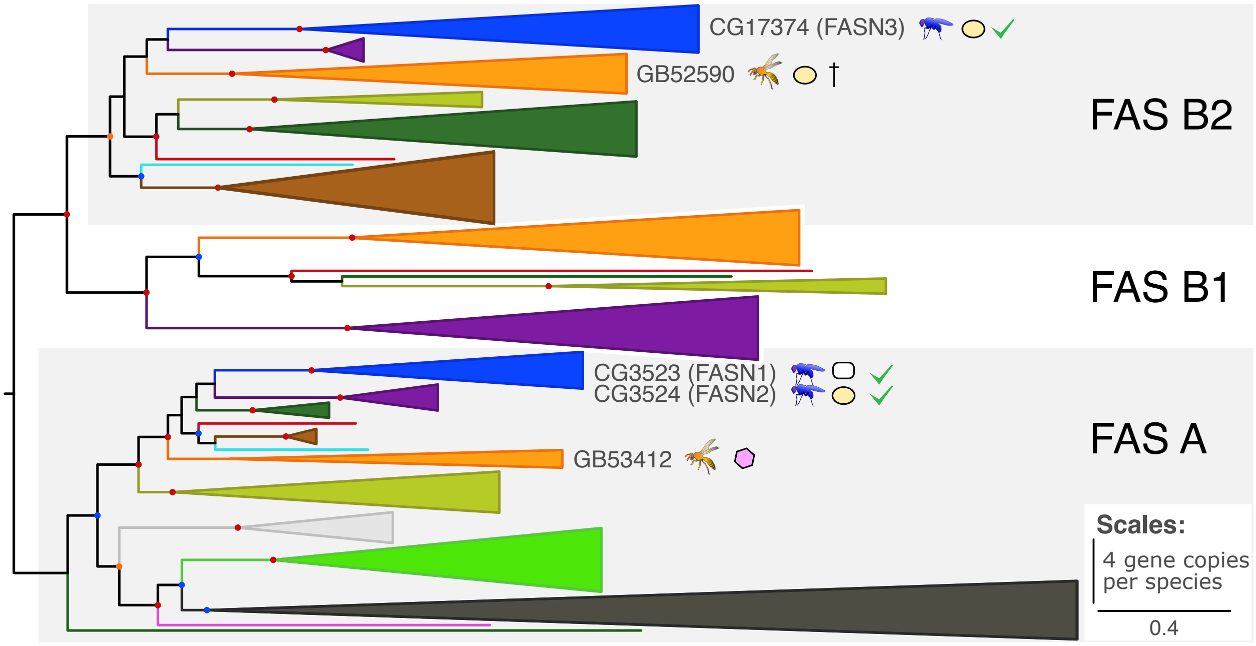

Color coding and symbols are as in the legend of ***Figure 2***, the cross indicates the target gene which knockdown caused a premature death of the treated bees.

***Supplementary Figure 3*. Gene tree of fatty acyl-CoA reductases from 37 species of Euarthropoda showing copy numbers and involvement of genes in CHC biosynthesis.**

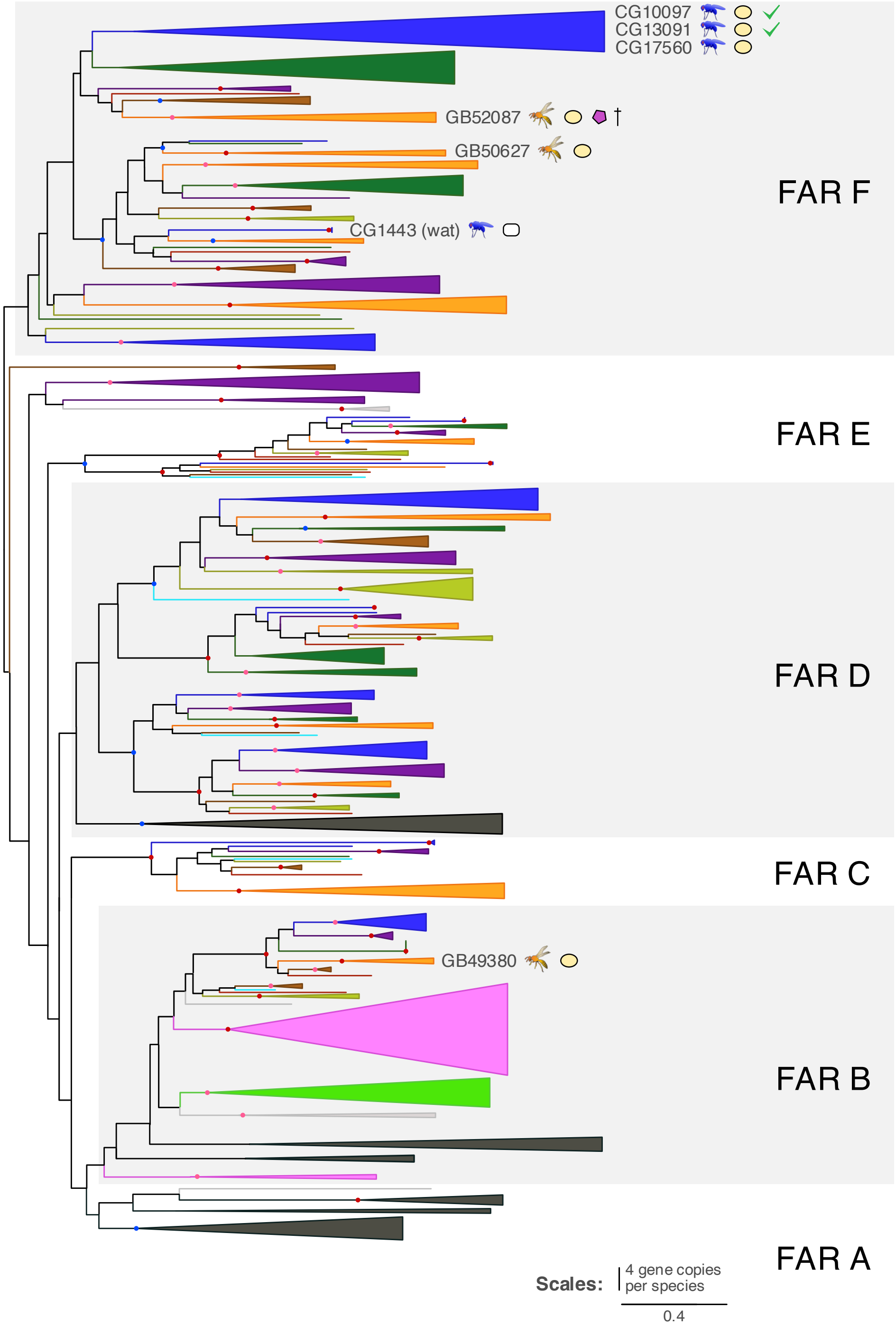

Color coding and symbols are as in the legend of ***Figure 2***, the cross indicates the target gene which knockdown caused a premature death of the treated bees.

***Supplementary Figure 4*. Composition photomicrographs of worker honey bee (*Apis mellifera*) fat body cells stained with control probes (sense RNA) of the candidate genes.**

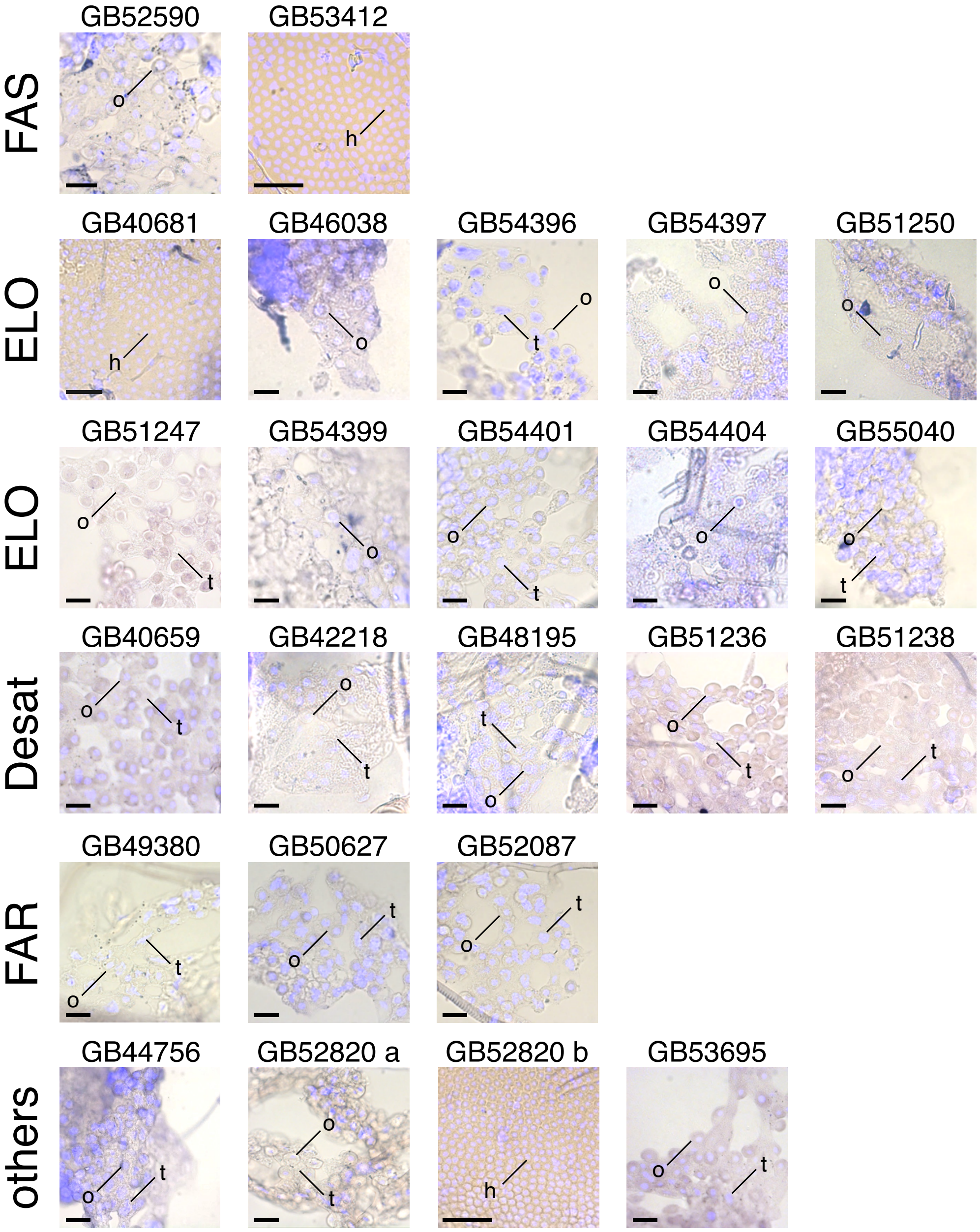

The cells are stained with DAPI (in light blue) and with the sense RNA probes of fatty acid synthases (FAS), elongases (ELO), desaturases (Desat), fatty acid reductases (FAR), and other candidate genes (others) in three types of cells: hexagonal cells (h), oenocytes (o), and trophocytes (t). DAPI was used to counterstain the nuclei of cells. Scale bar: 50 μm.

***Supplementary Figure 5*. Effects of RNAi-mediated knockdown of the fatty acid desaturase *GB51236* in worker honey bees (*Apis mellifera*) 2–5 days after dsRNA treatment.**

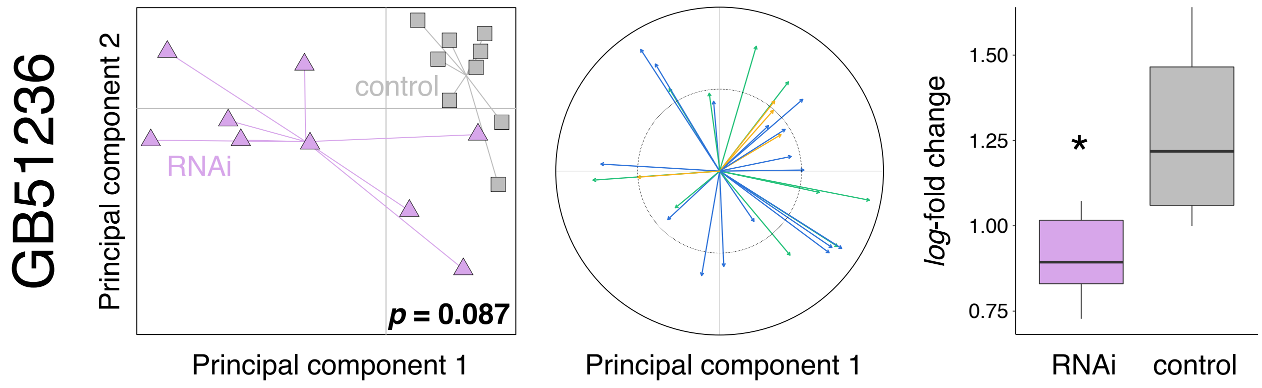

The first two columns show score plots and correlation circle plots from a powered partial least squares discriminant analysis (PPLS-DA) of cuticular hydrocarbon (CHC) profile data of target gene-treated (in purple) and of control bees (in grey, injected with dsRNA targeting GFP). *P*-values indicate the statistical probability of group differences representing random variation after Benjamini-Hochberg correction for multiple (N = 10) testing. The correlation circle plots indicate how many CHCs of a given compound class (i.e., alkanes [green], alkenes/alkadienes [blue], methyl-branched alkanes [yellow]) correlate with the first two principal components. Box plots in the third column show gene expression levels (*log_2*-fold change) of the target genes in target gene-treated (in purple) and in control bees (in grey). Asterisks indicate the statistical significance of expression differences between target gene-treated and control bees after applying Benjamini-Hochberg correction for multiple testing (N = 9; Welch's t-test/Wilcoxon signed-rank test; $p \leq 0.05$ [*], $p \leq 0.01$ [**]).

Supplementary Figure 6. **Correlation circle plots from a powered partial least squares discriminant analysis (PPLS-DA) of the cuticular hydrocarbon (CHC) profile data of target gene-treated bees (fatty acid elongases and fatty acid amide hydrolase) and of control bees.**

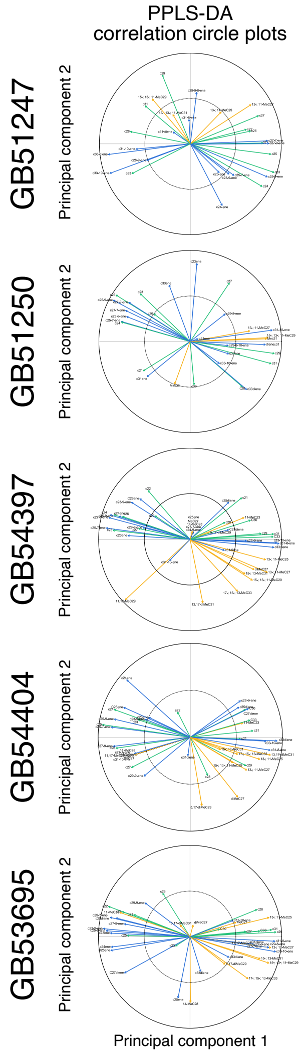

The correlation circle plots indicate how many CHCs of a given compound class (i.e., alkanes [green], alkenes/alkadienes [blue], methyl-branched alkanes [yellow]) correlate with the first two principal components separating the CHC profiles of RNAi-treated bees of the control bees in case of knockdown of four fatty acid elongases (*GB51247*, *GB51250*, *GB54397*, *GB54404*) and of one fatty acid amide hydrolase (*GB53695*) in worker honey bees (*Apis mellifera*) 2–5 days after dsRNA treatment. The specific identity of the CHCs is indicated at the tip of the arrows with established acronyms. The provided information complements that given in ***Figure 5***.

***Supplementary Figure 7.* Correlation circle plots from a powered partial least squares discriminant analysis (PPLS-DA) of the cuticular hydrocarbon (CHC) profile data of target gene-treated bees (fatty acid desaturases) and of control bees.**

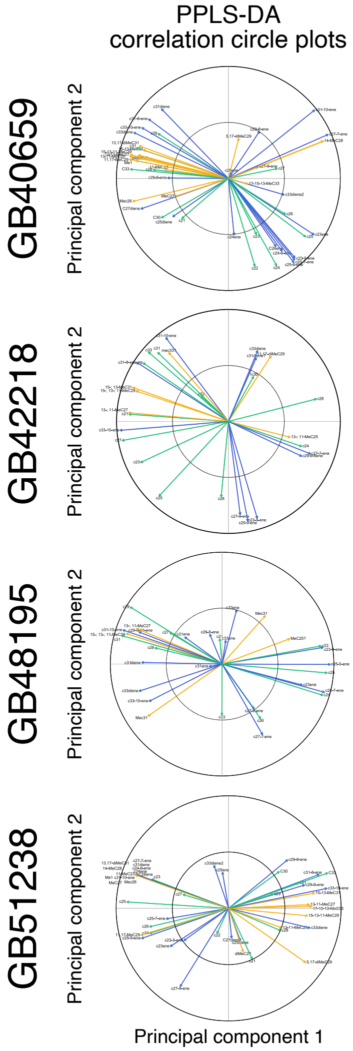

The correlation circle plots indicate how many CHCs of a given compound class (i.e., alkanes [green], alkenes/alkadienes [blue], methyl-branched alkanes [yellow]) correlate with the first two principal components separating the CHC profiles of RNAi-treated bees of the control bees in case of knockdown of four fatty acid desaturases (*GB40659*, *GB42218*, *GB48195*, *GB51238*) in worker honey bees (*Apis mellifera*) 2–5 days after dsRNA treatment. The specific identity of the CHCs is indicated at the tip of the arrows with established acronyms. The provided information complements that given in ***Figure 6***.

Supplementary Figure 8. **Gene tree of fatty acid amide hydrolases (FAAH) from 37 species of Euarthropoda showing copy numbers and involvement of genes in CHC biosynthesis**.

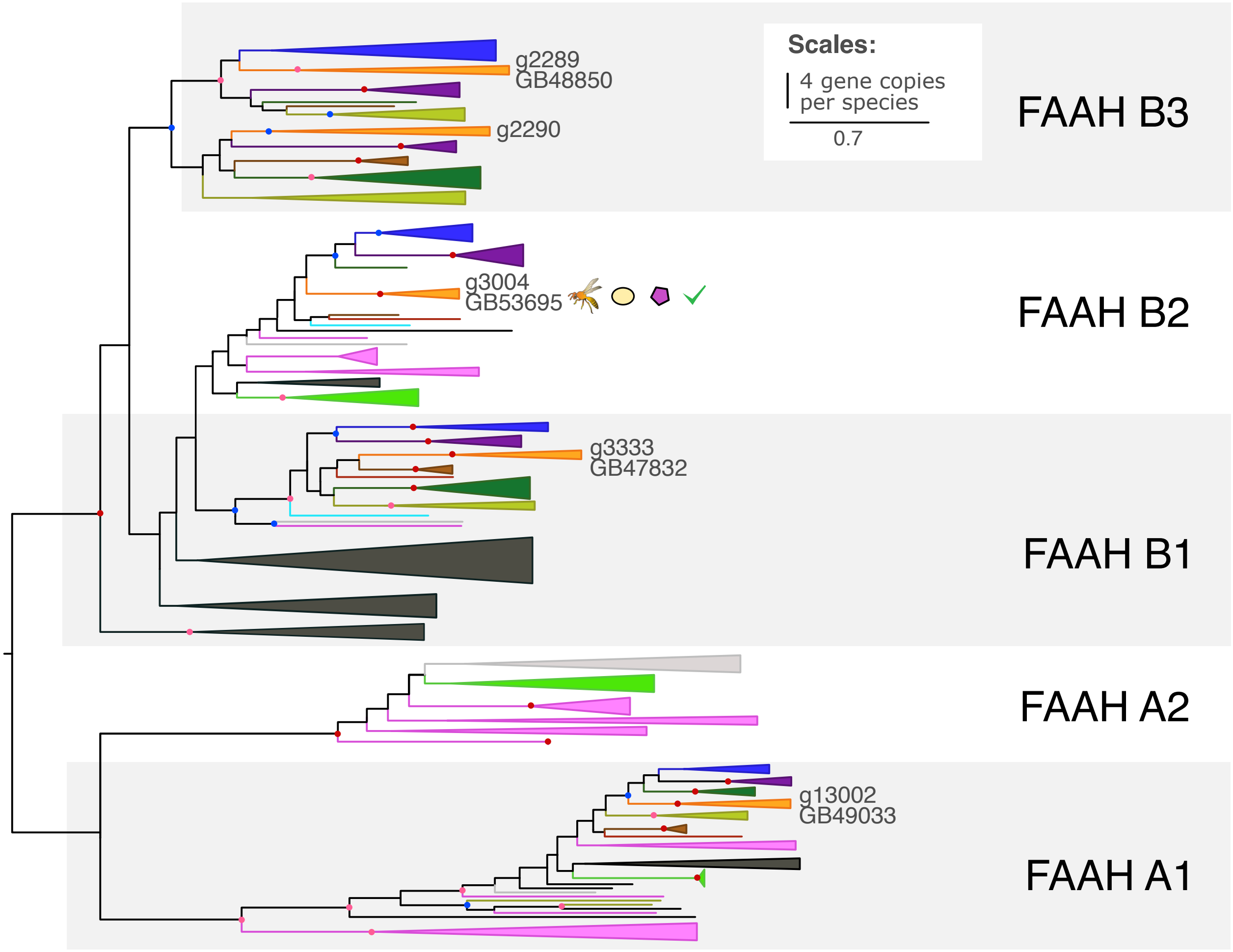

Color coding and symbols are as in the legend of ***Figure 2***.

Supplementary Figure 9. **Number of genes (y axis) found in the 37 species of Euarthropoda (x axis) for the five studied gene families: ELO (fatty acid elongase), Desat (fatty acid desaturase), FAR (fatty acyl-CoA reductase), FAS (fatty acid synthase), FAAH (fatty acid amide hydrolase).**

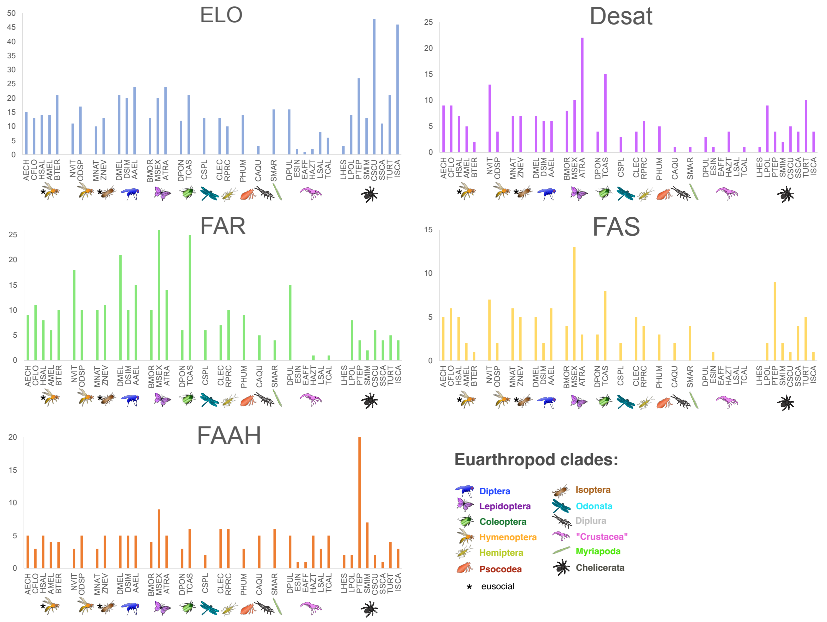

The 37 species represented are the following: eusocial Hymenoptera: AECH (*Acromyrmex echinatior*), CFLO (*Camponotus floridanus*), HSAL(*Harpegnathos saltator*), AMEL (*Apis mellifera*), BTER (*Bombus terrestris*), NVIT (*Nasonia vitripennis*); solitary hymenoptera: ODSP (*Odynerus spinipes*); Isoptera: MNAT (*Macrotermes natalensis*), ZNEV (*Zootermopsis nevadensis*); Diptera: DMEL (*Drosophila melanogaster*), DSIM (*Drosophila simulans*), AAEL (*Aedes aegypti*); Lepidoptera: BMOR (*Bombyx mori*), MSEX (*Manduca sexta*), ATRA (*Amyelois transitella*); Coleoptera: DPON (*Dendroctonus ponderosae*), TCAS (*Tribolium castaneum*); Odonata: CSPL (*Calopteryx splendens*); Hemiptera: CLEC (*Cimex lectularius*), RPRC (*Rhodnius prolixus*); Psocodea: PHUM (*Pediculus humanus*); Diplura: CAQU (*Catajapyx aquilonaris*); Myriapoda: SMAR (*Strigamia maritima*); “Crustacea”: DPUL (*Daphnia pulex*), ESIN (*Eriocheir sinensis*), EAFF (*Eurytemora affinis*), HAZT (*Hyalella azteca*), LSAL (*Lepeophtheirus salmonis*), TCAL (*Tigriopus californicus*); Chelicerata: LHES (*Latrodectus hesperus*), LPOL (*Limulus polyphemus*), PTEP (*Parasteatoda tepidariorum*), SMIM (*Stegodyphus mimosarum*), CSCU (*Centruroides sculpturatus*), SSCA (*Sarcoptes scabiei*), TURT (*Tetranychus urticae*), ISCA (*Ixodes scapularis*).
