# Supplementary material for: Intrasexual cuticular hydrocarbon dimorphism in a wasp sheds light on hydrocarbon biosynthesis genes in Hymenoptera
Source: Commun Biol. 2023 Feb 3;6:147. doi: 10.1038/s42003-022-04370-0 (PMC9898505; doi:10.1038/s42003-022-04370-0)
Supplement: Supplementary file 4 — Reporting Summary [file 42003_2022_4370_MOESM4_ESM.pdf]

nature portfolio

Corresponding author(s): Victoria C. Moris

Last updated by author(s): Nov 18, 2022

Reporting Summary

Nature Portfolio wishes to improve the reproducibility of the work that we publish. This form provides structure for consistency and transparency in reporting. For further information on Nature Portfolio policies, see our Editorial Policies and the Editorial Policy Checklist.

Statistics

For all statistical analyses, confirm that the following items are present in the figure legend, table legend, main text, or Methods section.

n/a	Confirmed	
☐	☒	The exact sample size (*n*) for each experimental group/condition, given as a discrete number and unit of measurement
☐	☒	A statement on whether measurements were taken from distinct samples or whether the same sample was measured repeatedly
☐	☒	The statistical test(s) used AND whether they are one- or two-sided<br>*Only common tests should be described solely by name; describe more complex techniques in the Methods section.*
☒	☐	A description of all covariates tested
☐	☒	A description of any assumptions or corrections, such as tests of normality and adjustment for multiple comparisons
☒	☐	A full description of the statistical parameters including central tendency (e.g. means) or other basic estimates (e.g. regression coefficient) AND variation (e.g. standard deviation) or associated estimates of uncertainty (e.g. confidence intervals)
☐	☒	For null hypothesis testing, the test statistic (e.g. *F*, *t*, *r*) with confidence intervals, effect sizes, degrees of freedom and *P* value noted<br>*Give P values as exact values whenever suitable.*
☒	☐	For Bayesian analysis, information on the choice of priors and Markov chain Monte Carlo settings
☒	☐	For hierarchical and complex designs, identification of the appropriate level for tests and full reporting of outcomes
☒	☐	Estimates of effect sizes (e.g. Cohen's *d*, Pearson's *r*), indicating how they were calculated

Our web collection on statistics for biologists contains articles on many of the points above.

Software and code

Policy information about availability of computer code

Data collection	software HP Enhanced Chem; Station G1701AA (version A.03.00; Hewlett Packard, Palo Alto, California, USA); GCSolution (Shimazu, Wemmel, Belgium); trimmomatic version 0.33; Platanus version 1.2.4; BRAKER ab initio gene prediction pipeline version 2.1; HISAT2 version 2.1.0; SAMtools version 1.7; BAMtools version 2.3.0; BRAKER2 pipeline (internally using GeneMark version 4.33);<br>Within Galaxy Europe platform (https://usegalaxy.eu): Trim Galore version 0.4.3.0 ; FastQC version 0.67 ; STAR version 2.5.2b0 ; featureCounts version 1.5.3; DESeq2 version 2.11.39<br>edgeR version 3.28.0; R statistical software version 3.4.1; Rstudio (R version 4.0.3); PFAM database (PFAM-A, version 28); hmmer version 3.1b1; MAFFT version 7.123; IQ-TREE version 1.6; BLAST+ software suite; Primer-BLAST; ImageJ; FigTree version 1.4.3; Interactive Tree of Life (ITOL) version 3 online tool; FlyAtlas2; Inkscape version 1.1
Data analysis	RVAideMemoire version 0.9-80; vegan version 2.5-7; Hotelling version 1.0-7; pls version 2.7-3; FactoMineR version 2.4; Factoextra version 1.0.6; stats package version 3.6.2

For manuscripts utilizing custom algorithms or software that are central to the research but not yet described in published literature, software must be made available to editors and reviewers. We strongly encourage code deposition in a community repository (e.g. GitHub). See the Nature Portfolio guidelines for submitting code & software for further information.

Data

Policy information about availability of data

All manuscripts must include a data availability statement. This statement should provide the following information, where applicable:

- Accession codes, unique identifiers, or web links for publicly available datasets
- A description of any restrictions on data availability
- For clinical datasets or third party data, please ensure that the statement adheres to our policy

The nucleotide sequence data of the genome shotgun project are deposited under the NCBI Bioproject PRJNA735081. The draft genome assembly and the corresponding gene annotations are available under the NCBI accession JAIFRP000000000 and from Zenodo (10.5281/zenodo.5552394). The transcriptomic raw reads are available from GenBank in Bioproject PRJNA609595. Additional data that support the findings of this study have been deposited on Zenodo (10.5281/zenodo.5552394).

Field-specific reporting

Please select the one below that is the best fit for your research. If you are not sure, read the appropriate sections before making your selection.

☐ Life sciences ☐ Behavioural & social sciences ☒ Ecological, evolutionary & environmental sciences

For a reference copy of the document with all sections, see nature.com/documents/nr-reporting-summary-flat.pdf

Ecological, evolutionary & environmental sciences study design

All studies must disclose on these points even when the disclosure is negative.

Study description	We performed chemical analyses of wasps in the field and in the laboratory, we also analyzed the differential gene expression with transcriptomic sequencing of Odynerus spinipes females. We used honey bee workers for in situ hybridization and knockdown experiments.
Research sample	We used Odynerus spinipes females to evaluate if they change their chemotype during their lifespan and to study the genes differentially expressed in the two chemotypes and find candidate genes.<br>We used Apis mellifera workers to perform in situ hybridization and knockdown experiments to avoid performing these on O. spinipes which is found in the field in small sample size.
Sampling strategy	We used 6 O. spinipes females of each chemotype to perform the differential gene expression analyses. Such analyses can be performed already with only three replicates of each condition. By doubling the number of required number of replicates we assured high confidence in our analysis.<br>We sampled the CHC profile of 23 O. spinipes females in the laboratory and of 18 females in the field. With these number of replicates, we provided strong evidence that O. spinipes females do not change their chemotype during their lifespan.<br>While performing the knockdown experiments, we injected 10 to 15 bees with dsRNA of target genes and 10 to 15 bees with dsRNA of GFP, serving as controls. Since the injection process sometimes killed the bees because of the needle, we started the experiment with 10-15 bees per treatment in order to have at the end at least 6 replicates per treatment which allowed us to perform statistical analyses.
Data collection	The CHCs were extracted from the bees and wasps with the animals being anesthesized (cold or CO_2 exposure) by V. Moris using SPME fibers. When it was needed to also analyze the gene expression level (for qPCR in case of knockdown experiments), V. Moris euthanized them extracting their CHCs in hexane and freeze-killing them in liquid nitrogen. Bees used for in situ hybridization were euthanized by V. Moris by freeze-killing them in liquid nitrogen.
Timing and spatial scale	CHC sampling on O. spinipes females in the laboratory was performed in April and May 2016, the sampling in the field with marked wasps was performed during the season where we could find the wasps in the field: end of May until July in 2016 and 2017. Sampling of O. spinipes females in the field at two different locations (Büchelberg (49.027985, 8.164801)and Tenneville (50.100671, 5.530061) allowed to confirm if we observed the same results in two different populations. It also allowed to reduce the stress we might have caused (by capturing the females, marking them, sampling their CHCs and releasing them) on a single population.<br>The three genomic DNA NGS libraries were built from a single pool of DNA extracted from meso- and metasomas of 10 males (collected May 15 and 16, 2013) and 31 females (collected May 15 and 16, 2013) of Odynerus spinipes collected near Würzburg, Germany (49.77977, 9.97065). The transcriptomes of one O. spinipes male and two females collected in Büchelberg in 2014 were used to facilitate annotation of the Odynerus spinipes draft genome.<br>O. spinipes females used for transcriptomic sequencing were sampled from the field in Büchelberg (49.027985, 8.164801), Bad Muskau (51.548200, 14.714900), and Eichenzell (50.495927, 9.704589) through different years (2015, 2016, 2017).<br>In situ hybridization and knockdown experiments were carried out during two summers (2018 and 2019) using honey bee workers (from bee hives at the University of Würzburg) in order to test high number of candidate genes. Since negative controls were performed for each knockdown experiments, performing these experiments during different years did not impacted the analyses.
Data exclusions	The only data that we excluded from the analysis were peaks from the CHC extracts which were due to column bleeding, or peaks that were present only in traces (< 0.3 % of the total abundance), or peaks other than CHCs.
Reproducibility	The continous sampling of CHC of O. spinipes females in the field was performed at two different field sites and also at two different years and confirmed the results that females do not change their chemotype during their life.

In case of the differential gene expression analysis using O. spinipes transcriptomes, we first analyzed one batch of three females of each chemotype aged of 12-38 hours. In order to confirm the candidate genes found, we sequenced a second batch of females. Since we found that O. spinipes females, right after their eclosion, have low quantities of CHCs on their cuticle, we sampled O. spinipes females of the second batch at a different age (3-days-old). We sampled them from the same field site and two other field sites Eichenzell (50.495927, 9.704589) and Bad Muskau (51.3254, 14.4253) to avoid identifying genes as candidates which are not chemotype-specific but specific from the Bûchelberg population.
We performed the in situ hybridization experiments with multiple honey bee metasoma tergites and sternites and observe the signal on each of them.

Randomization	Randomization was not relevant in this study. It will not interfere with the continous sampling of O. spinipes females in the field and in the laboratory, nor for the differential expression analysis. In case of the in situ hybridization experiments, randomization is also not required. We might have benefit from it while analyzing the CHC profiles of dsRNA treated honey bees for the knockdown experiments. However, it was not logistically possible. Running the CHC extracts with the GC-MS in a randomized order would have taken more time. We would have to wait for all the experiments to be carried out, and then attribute a number to all CHC extracts to run them in a random order. We do not think that our analyses suffer from the non-randomization of the order in which the CHC extracts were processed in the GC-MS. We carefully analyzed the CHC profile, verifying for column bleeding, and removed peaks other than CHCs in order to focus on the effect of RNA interference on CHCs.
Blinding	CHC extracts from O. spinipes females or A. mellifera were identified by a number. Therefore, while analyzing each profile individually, we never knew which condition was being analyzed which allowed to process them without being biased.<br>Regarding the in situ hybridization, we used as negative controls, sense probes. While performing the staining, we only verified after a staining was observed in one of the two conditions (antisense and sense) if it corresponded to the sense or antisense probe which allowed to not be biased while observing the staining process.

Did the study involve field work? ☒ Yes ☐ No

Field work, collection and transport

Field conditions	We were sampling in the field during the O. spinipes season (late March until beginning of July) whenever it was not raining.
Location	O. spinipes prepupae were sampled in Büchelberg (49.027985, 8.164801), Bad Muskau (51.548200, 14.714900), Eichenzell (50.495927, 9.704589), and Würzburg (49.77977, 9.97065), where were placed selfmade trap nests.<br>CHC sampling of O. spinipes in the field was performed in Büchelberg, in Germany (49.027985, 8.164801) and Tenneville, in Belgium (50.100671, 5.530061).
Access & import/export	Trap nests were placed in the private garden of Wolf-Harald Liebig in Bad Muskau and Karl-Heinz Schmalz in Eichenzell. They were carefully removed and opened in order to find prepupae.<br>We received a permit for collecting samples in Büchelberg by the Struktur- and Genehmigungsdirektion Süd from 01.03.2016 to 31.12.2019 and permission to place trap nests at a bee hotel in Büchelberg by Gaby Schöning.<br>The nesting site in Büchelberg is close to a way taken by agricultures and was therefore easily accessible by car.<br>The location in Tenneville was given by Jean-Yves Baugnée. A permit was not required to collect O. spinipes there.<br>V. Moris accessed the nesting site in Tenneville by foot in order to avoid causing disturbances in the landscape.
Disturbance	We placed a few selfmade trap nests in Büchelberg. They consisted of 20 cm long plastic pipes with a clear diameter of 10 cm filled with eolian silt deposit. The eolian silt deposit contained pre-built 7 cm long channels with a diameter of 0.6 cm each. The trap nests were mounted on posts at a height between 5 and 100 cm from the ground in Büchelberg. We placed them close to the bee hotel which was present in Büchelberg so they would not disturb the landscape. Since it was not a natural nest site for O. spinipes, the trap nests placed there for several years were likely helping the population providing a good nesting site.<br>The population in Tenneville was more important than in Büchelberg which allowed us to sample several wasps without impacting the population.<br>The CHC sampling in the field was done in a way to minimize the impacts on the population by using color markers that did not disturb them and by sampling their CHCs in a non invasive way, using SPME fibers. The marked wasps were observed during a long period of time and were able to continue building their nests and providing them with food.

Reporting for specific materials, systems and methods

We require information from authors about some types of materials, experimental systems and methods used in many studies. Here, indicate whether each material, system or method listed is relevant to your study. If you are not sure if a list item applies to your research, read the appropriate section before selecting a response.

Materials & experimental systems

n/a	Involved in the study	
☒	☐	Antibodies
☒	☐	Eukaryotic cell lines
☒	☐	Palaeontology and archaeology
☐	☒	Animals and other organisms
☒	☐	Human research participants
☒	☐	Clinical data
☒	☐	Dual use research of concern

Methods

n/a	Involved in the study	
☒	☐	ChIP-seq
☒	☐	Flow cytometry
☒	☐	MRI-based neuroimaging

Animals and other organisms

Policy information about studies involving animals; ARRIVE guidelines recommended for reporting animal research

Laboratory animals	NA
Wild animals	Odynerus spinipes females and males were captured in the field for genomic sequencing. O. spinipes prepupae were captured in the field and were raised in the laboratory and then euthanized to sequence their transcriptome (12-62 hours after eclosion). O. spinipes prepupae were captured in the field to be raised in the laboratory for their whole life and to sample at multiple times their CHC profiles. O. spinipes females were captured, marked and released in the field in order to sample their CHC profiles at multiple times of their lifespan.<br>Apis mellifera prepupae workers from bee hives at the University of Würzburg were collected and raised in the laboratory in a incubator in the dark at 34 °C. They were euthanized ca. 10 days after eclosion.
Field-collected samples	O. spinipes females used for transcriptomic sequencing were kept separate from each other at 21 °C in polystyrene tubes (53 mm x 100 mm, Bioform, Nuremberg, Germany) containing moistened cotton and experienced a 12/12 h day/night cycle.<br>Each O. spinipes female raised in the laboratory to sample their CHC at multiple times, was placed in a separate observation cage (30 cm x 30 cm x 30 cm, Bioform, Nürnberg, Germany) and provided with a conspecific male and absorbent paper soaked with honey and water. The presence of males was meant to simulate field conditions for the females. The cages were kept in a climate chamber (70% humidity, 23 °C during day, 18 °C during night, and with a 12/12 h day/night cycle).<br>A. mellifera workers came from the same hive, eclosed 6–7 days prior to dsRNA treatment and belonged to the same caste (workers, nurse bee). Bees of different experiments were kept in separated observation cages and were fed with fed 30% sugar water.
Ethics oversight	NA

Note that full information on the approval of the study protocol must also be provided in the manuscript.
